# Regulatory effects of exogenous calcium on sweet cherry fruit cracking and functional analysis of the *PavCML42* gene

**DOI:** 10.3389/fpls.2026.1916674

**Published:** 2026-08-13

**Authors:** Huijuan Yang, Sen Hou, Shuaibing Jiang, Guoqin Wei, Junping Wu, Shengnan Zhu, Miroslaw Sitarek, Quanjuan Fu

**Affiliations:** 1 Shandong Center of Technology Innovation for Modern Protected Fruit Tree./Shandong institute of Pomology, Taian, Shandong, China; 2 College of Horticulture Science and Engineering. Shandong Agricultural University, Taian, Shandong, China; 3 Tai’an Forestry Protection and Development Center, Taian, China; 4 Department of Cultivars Testing, Nursery and Genetic Resources, The National Institute of Horticultural Research, Skierniewice, Poland

**Keywords:** CML gene, exogenous calcium, fruit cracking, sweet cherry, transcriptome

## Abstract

Rain‑induced fruit cracking during sweet cherry ripening severely restricts industry development, but whether calcium signaling regulates cracking through CML genes remains unclear. Using the crack‑prone cultivar ‘Brooks’, we investigated the physiological and molecular mechanisms of calcium‑mediated regulation through exogenous CaCl_2_ spraying, physiological index measurements, transcriptome analysis, and gene function validation. Treatment with 0.5% CaCl_2_ was most effective, reducing the cracking rate by 53.3%, decreasing MDA content, modulating antioxidant enzyme activities, and inhibiting cell wall hydrolase activities. Transcriptome analysis identified 18 calcium‑signaling‑related DEGs, among which *PavCML42* showed the greatest differential expression. Tissue‑specific expression analysis revealed highest expression in roots and lowest in fruit. Transient overexpression of *PavCML42* significantly increased cell wall hydrolase activities and induced cracking, whereas VIGS silencing reduced these enzyme activities and alleviated membrane lipid peroxidation damage. Overall, exogenous calcium mitigates fruit cracking by regulating *PavCML42*, which is involved in cell wall metabolism and the antioxidant system, providing a candidate gene for breeding crack‑resistant sweet cherry cultivars.

## 1 Introduction

Sweet cherry (*Prunus avium* L.) is a perennial temperate deciduous fruit tree in the Rosaceae family. It is currently commercially cultivated in more than 40 countries and is an important global economic crop. In China, the planting area and yield of sweet cherries have continued to grow, with Shandong becoming the country’s largest sweet cherry-producing region (Liu et al., 2022). Sweet cherry fruits are rich in anthocyanins, hydroxycinnamic acids, and other polyphenolic compounds, and have various health benefits, including antioxidant, anti-inflammatory, and blood glucose–regulating effects (Blando and Oomah, 2019), making them popular with consumers. However, cherries are highly prone to fruit cracking when it rains during the ripening period; cracking rates in susceptible cultivars can reach as high as 90%, and annual economic losses due to cracking are about 30%, reaching up to 80% in severe cases, which seriously affects fruit quality and market value and limits industry development (Correia et al., 2018). Sweet cherry fruit cracking not only affects edibility and reduces fruit quality and yield, but cracked sites are also more easily infected by pathogenic microorganisms and facilitate pest and disease spread, further reducing economic returns (Niu et al., 2020).

Fruit cracking is a physiological disorder caused by the inability of fruit growth and development to adapt to the external environment conditions, resulting in cracking of the peel or even the flesh (Brüggenwirth and Knoche, 2017). This phenomenon commonly occurs in horticultural plants such as jujube (Li et al., 2019), lychee (Wang et al., 2019), sweet cherry (Schumann et al., 2019), grape (Lang and Düring, 2015), and tomato (Jiang et al., 2019). The occurrence of fruit cracking is closely related to factors including water stress during fruit ripening, changes in cell wall structure, and imbalances in reactive oxygen species metabolism, but its molecular regulatory mechanisms remain incompletely understood. Calcium ions (Ca^2+^), as a second messenger in plant signal transduction, are one of the effective regulators of cellular metabolism and can participate in responses to various stresses such as low temperature, high temperature, salt stress, drought, and mechanical stimulation (Medvedev, 2005). During plant growth and development, Ca^2+^ can not only be involved in cell division and differentiation to regulate various growth stages, but also form calcium bridges with pectic acids to enhance cell wall stability and regulate membrane permeability (Bangerth, 1979). Foliar calcium application is an effective measure for preventing fruit cracking. As early as 1937, it was found that spraying Bordeaux mixture could significantly reduce fruit cracking after rainfall. Since then, various calcium formulations-including calcium acetate, calcium nitrate, and calcium chloride- have been widely used to improve fruit quality and control cracking, with their efficacy varying depending on calcium type, application timing, and crop species (Callan, 1986). Recent studies have shown that spraying calcium during the young fruit and fruit enlargement stages significantly increase peel calcium content and firmness, and reduces cracking rates in sweet cherry (Qu et al., 2014), lychee (Peng et al., 2001), and various fruit trees (Chen et al., 2014; Guo et al., 2019).

Ca^2+^ signals require transduction by calcium-binding proteins. Currently, four main classes of calcium-binding proteins are recognized in plants: calmodulins (CaM), calmodulin-like proteins (CML), calcium-dependent protein kinases (CDPKs), and calcineurin B-like proteins (CBL) (Poovaiah et al., 2013; McCormack and Braam, 2003; Weinl and Kudla, 2009; Schulz et al., 2013). All four families of calcium-binding proteins contain highly conserved domains that can rapidly bind or release Ca^2+^, responding to and regulating changes in intracellular Ca^2+^ concentration (Tidow and Nissen, 2013). Among them, CML is a plant-specific subfamily of calcium-binding proteins. 50, 52, 54, 32, and 23 members have been identified in Arabidopsis, tomato, melon, rice, and jujube, respectively (McCormack and Braam, 2003; Munir et al., 2016; Luo et al., 2021; Boonburapong and Buaboocha, 2007; Gao et al., 2023). CML proteins have 1–6 conserved EF-hand domains, share at least 16% amino acid similarity with CaM, and do not contain other functional domains (McCormack and Braam, 2003), indicating that, as primary responders to calcium signals, they likely participate mainly in signal transduction rather than directly executing functions.

Functional studies show that CML family members play important roles in plant growth and in biotic and in responses to biotic and abiotic stresses. In Arabidopsis, *AtCML7* regulates root hair elongation (Won et al., 2009); *AtCML9* is involved in responses to salt, drought, and ABA stress (Magnan et al., 2008); *AtCML18* enhances salt tolerance by binding the target protein NHX1 (Yamaguchi et al., 2005); *AtCML25* regulates K^+^ influx, affecting pollen germination rate and pollen tube elongation (Wang et al., 2015); *AtCML42* suppresses ABA biosynthesis in response to drought stress (Vadassery et al., 2012). In other species, jujube *CML13* responds to low-temperature stress (Gao et al., 2023); tea tree *CsCML16*, *CsCML18-2*, and *CsCML42* respond to salt stress (Ma et al., 2019); rice *OsCML4* and *OsMSR2* can resist drought and salt stresses (Xu et al., 2011; Yin et al., 2015).

Recent studies have found that CML gene expression levels are significantly higher at the cracking sites of jujube and sand pear than in normal fruit (Wang et al., 2021; Fan et al., 2023), indicating that the CML family may be involved in regulating fruit cracking. Although exogenous calcium spraying has been shown to reduce fruit cracking rates in various fruits, it remains unclear whether calcium signaling regulates sweet cherry cracking via CML genes and what the underlying physiological and molecular mechanisms are. Based on this, the present study used the crack-prone sweet cherry cultivar ‘Brooks’ as experimental material to investigate the mechanism by which calcium regulates cracking. Through exogenous calcium spray treatments combined with physiological measurements, transcriptome analysis, and gene function validation, we aimed to identify candidate CML genes responsive to calcium signaling and clarify their functions.

## 2 Materials and methods

### 2.1 Plant materials

The experiment was conducted at the experimental station of the Shandong Institute of Pomology. The plant material were 9-year-old sweet cherry cultivar ‘Brooks’ grafted onto rootstock ‘Gisela 6’. The orchard was planted using cultivation with wide rows and high density; trees were trained to a slender spindle form and managed with conventional water and fertilizer practices.

The tomato cultivar used was ‘Millennium’. One hundred fruits uniform in maturity and size, and free from visible pests and diseases were selected and retained.

### 2.2 Experimental treatments and sampling

#### 2.2.1 Calcium treatment

Sweet cherry trees of the ‘Brooks’ variety exhibiting uniform growth were selected for foliar spraying with CaCl_2_ solution at 45 days after flowering. Five concentration gradients were established: 0%, 0.25%, 0.5%, 0.75%, and 1% CaCl_2_. Spraying was conducted once every 48 hours for three consecutive applications, at a spraying height of 1–1.5 m targeting the canopy. The parameters were set according to previously reported methods (Zou et al., 2024; Matteo et al., 2024). After the treatment, fruits of uniform size and free from visible pests and diseases were collected from the sprayed areas and transported to the laboratory for subsequent analysis. For each treatment, three biological replicates were performed. From each replicate, 100 fruits were taken for cracking rate determination, and 10 fruits were randomly selected from these 100 for physiological assays.

#### 2.2.2 Determination of fruit cracking rate

Following the method of Winkler et al. (2020), 100 fruits of uniform size and free from damage were selected and submerged in tap water to simulate rainfall conditions and induce cracking. After 4 hours, the number of cracked fruits was counted, and the fruit cracking rate was calculated. Each treatment was replicated three times.


Fruit cracking rate (%) = (Number of craked fruits/Total number treated fruits ) × 100

#### 2.2.3 Sample collection for transcriptome sequencing

Five treatments were set up for transcriptome sequencing: (1) CK: untreated normal fruits; (2) CK-B: uncracked fruits sprayed with water; (3) CK-YL: the cracked tissue from fruits sprayed with clear water; (4) T-B: uncracked fruits sprayed with 0.5% CaCl_2_; (5) T-YL: the cracked tissue from fruits sprayed with 0.5% CaCl_2_. A 2-mm thick layer was sampled from the fruit surface, and three biological replicates were performed per treatment. After collection, the samples were immediately frozen in liquid nitrogen and stored at -80°C until further analysis.

### 2.3 Measurement of physiological parameters

Peel and pulp were separately collected from untreated normal fruits and cracked fruits for the determination of malondialdehyde (MDA) and antioxidant enzymes (SOD, POD, CAT, PPO), pectin content (protopectin [PP] and soluble pectin [SP]), and cell wall hydrolase activities (PG, CX, PME). Additionally, fruits treated with different concentrations of CaCl_2_ were submerged in water for 4 hours to induce cracking, after which the peel and pulp were similarly separated for the determination of the aforementioned parameters. Positive and negative controls obtained from transient overexpression and gene silencing injection experiments were also subjected to the same measurements. Each treatment was replicated three times, with 10 fruits used per replicate. SOD activity was determined using the nitroblue tetrazolium (NBT) photochemical reduction method (Cao et al., 2014). The activities of POD, CAT, and PPO, as well as malondialdehyde (MDA) content, soluble pectin (SP) and protopectin (PP) contents, were measured according to the method of Cao et al. (2007). PG and CX activities were assayed using commercial kits (Boxbio, Beijing, China), and PME activity was determined using a PME activity assay kit (Addison, China) by titration.

### 2.4 RNA extraction and gene expression analysis

Total RNA was extracted from the fruits using the Plant Polysaccharide and Polyphenol Total RNA Extraction Kit from Beijing Nobele Biochemical Technology Co., Ltd. Reverse transcription was performed using a kit from Beijing Quanshijin Biotechnology Co., Ltd. *PavActin* was used as an internal control, and qRT-PCR was conducted using the 2× SYBR Green Master Mix from AG Aicrui Biotechnology Co., Ltd. Relative expression levels were calculated using the 2^–ΔΔCT^ method, with three technical replicates performed for each sample. All primers used for qRT-PCR and vector construction are listed in **Supplementary Table 1**. Transcriptome sequencing was performed by Beijing Novogene Technology Co., Ltd. using the Illumina HiSeq™ sequencing platform. The raw sequencing data have been deposited in the NCBI Sequence Read Archive (SRA) under accession number PRJNA1493284. The RNA-seq data quality statistics are summarized in **Supplementary Table 2**.

### 2.5 Subcellular localization

After transferring the constructed pZP211-GFP-*PavCML42* vector into Agrobacterium, single colonies were picked and cultured with shaking. The resulting cells were resuspended in MMA buffer (10 mmol/L MgCl_2_, 10 mmol/L MES, 200 mmol/L AS), adjust the OD to 0.8–1.0 using a UV spectrophotometer, incubate at room temperature in the dark for 3 h. First, pierce the underside of a tobacco leaf with a 1 mL sterile syringe needle; then, remove the needle, draw up the suspension, The infiltration volume was controlled to produce an infiltrated area of approximately 1 cm in diameter to avoid excessive injection that may cause non-specific localization. Subsequently, the infiltrated leaves were incubated in the dark for 24 h, followed by 1 day of incubation under normal light conditions, and then observe the fluorescence using a two-photon confocal microscope.

### 2.6 Transient expression of the target gene in sweet cherry fruits mediated by a viral vector

The target gene fragment was cloned into the IL60-2 viral expression vector to obtain a recombinant plasmid. IL60-1 (helper vector), the IL60-2 empty vector, and the IL60 recombinant vector were each transformed into Agrobacterium tumefaciens GV3101; after PCR verification, they were used in subsequent experiments. The positive Agrobacterium colonies were cultured for activation and resuspended in 10 mM MgCl_2_ to a concentration of 10 ng/μL. The recombinant vector or the IL60-2 empty vector was mixed with IL60-1 at a 1:5 ratio to prepare the infiltration solution. Tomato fruits and detached sweet cherry fruits of uniform size were selected. The infiltration solution was injected into the fruit surface after the surface had been lightly pricked with a 1 mL syringe needle. Following injection, the fruits were incubated in the dark for 24 hours, then transferred to normal light conditions to observe phenotypic changes and collect samples for subsequent analysis.

### 2.7 VIGS-mediated gene silencing of *PavCML42* in sweet cherry

Glycerol stocks of Agrobacterium containing TRV2-*PavCML42*, TRV2-empty vector, and TRV1 helper vector were revived, and the cells were collected by centrifugation at 4,500 rpm and resuspended in MMA solution (10 mmol/L MgCl_2_, 10 mmol/L MES, and 200 µmol/L AS). A 1:1 mixture of TRV1 and TRV2-empty vector was used as the negative control, and a 1:1 mixture of TRV1 and TRV2-*PavCML42* was used as the silencing treatment. The mixtures were incubated in the dark for 30–60 min before infiltration. Sweet cherry fruits were lightly pricked with a 1 mL syringe needle and infiltrated with the bacterial suspensions. After infiltration, the fruits were kept in the dark for 48 h and then transferred to normal light conditions. Phenotypic observation and sample collection were performed on the second day post-infiltration. The samples were immediately frozen in liquid nitrogen and stored at –80°C. The expression level of *PavCML42* was determined by qRT-PCR, and the silencing efficiency was calculated as:


Silencing efficiency (%)= (Expression level in control−Expression level in treatment)/Expression level in control×100%

### 2.8 Statistical analysis

All experiments were performed with three replicates, data are expressed as mean ± standard error (SE), and significance tests (*P* < 0.05) were conducted using SPSS 16.0 software. Figures were generated using Excel and Graphpad prism. Accordingly, we have revised all figures by adding error bars and lowercase letter annotations to indicate significant differences among treatments.

## 3 Results and analysis

### 3.1 Effects of fruit cracking on the physiological characteristics of sweet cherry fruits

**Figure 1:**
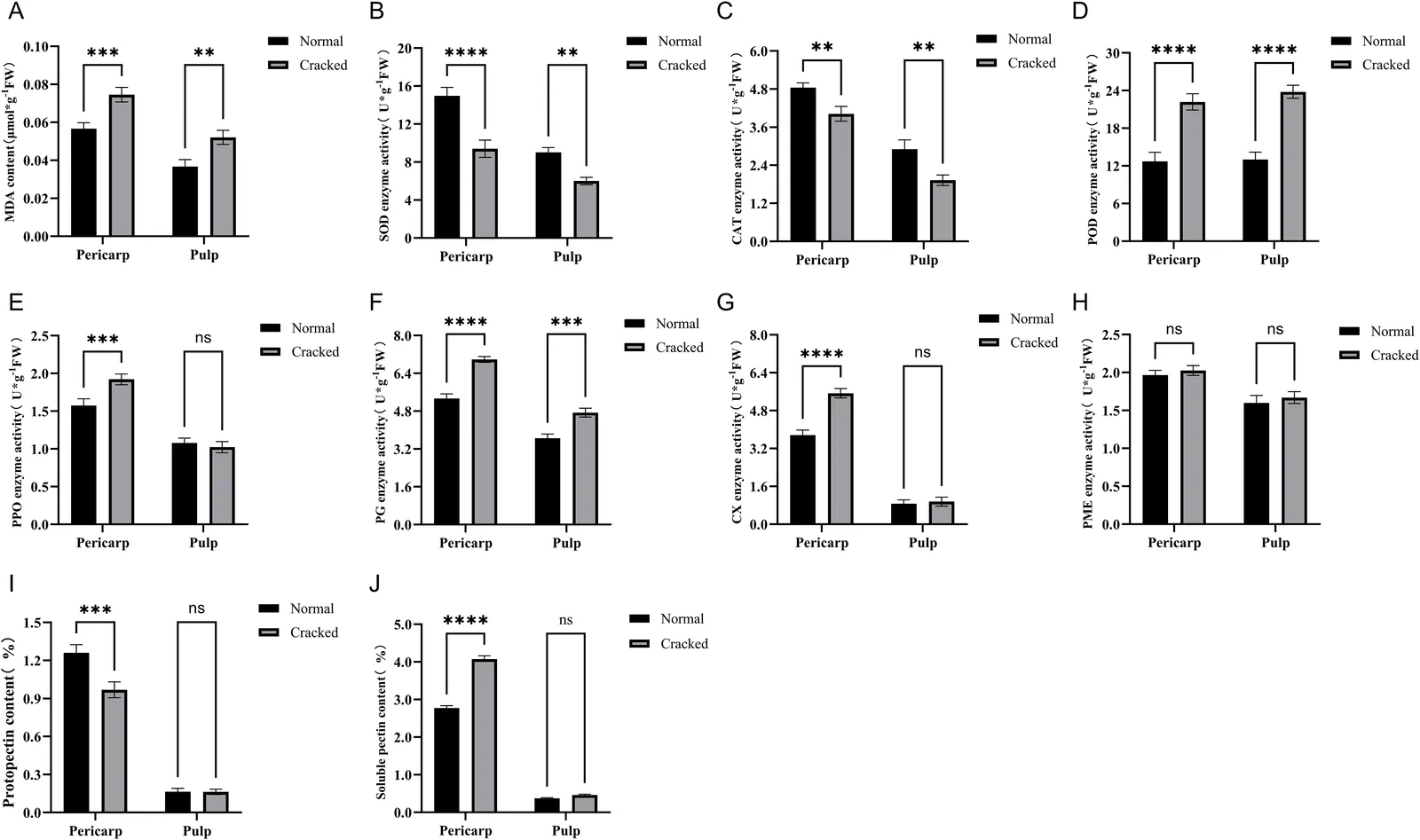
Physiological index differences between cracked fruits and normal fruits. Data are presented as mean ± SE. Statistical significance was determined by Student's t-test. * indicates *P*<0.05, *P*<0.05, ** indicates *P*<0.01, *** indicates *P*<0.001, **** indicates *P*<0.0001, and ns indicates not significant (*P*≥0.05).

To clarify the relationship between sweet cherry fruit cracking and fruit physiological metabolism, antioxidant enzyme activities and the activities of cell wall-metabolism-related substances were measured in cracked and normal fruits. The results showed that MDA contents in the flesh and peel of cracked fruits were significantly higher than those of normal fruits, increasing by 31.6% and 41.7%, respectively, and MDA content in the peel was greater than in the flesh. In terms of antioxidant enzyme activities, SOD activities in the peel and flesh of normal fruits were 59.4% and 20.5% higher than those of cracked fruits, respectively, while CAT activities in the peel and flesh of normal fruits were 50.3% and 50.6% higher, respectively. In contrast, POD and PPO activities showed the opposite trend. POD activities in the peel and flesh of cracked fruits were 74.7% and 82.9% higher than those of normal fruits, respectively; PPO activity in the peel of cracked fruits was 22.1% higher than that of normal fruits, while no significant difference in enzyme activity differences in the flesh were not significant. Assays of cell wall-hydrolyzing enzymes showed that PG and CX activities in the peel of cracked fruits were significantly higher than those in normal fruits, with increases of 30.9% and 46.9%, respectively. Correspondingly, PP content in the peel of cracked fruits was significantly lower than in normal fruits, while SP content was significantly higher (**Figure 1**). These results indicate that fruit cracking is closely related to aggravated membrane lipid peroxidation, an imbalance of the antioxidant system, and the degradation of cell wall pectin.

### 3.2 Measurement of physiological indicators of sweet cherry under different CaCl_2_ concentrations

**Figure 2:**
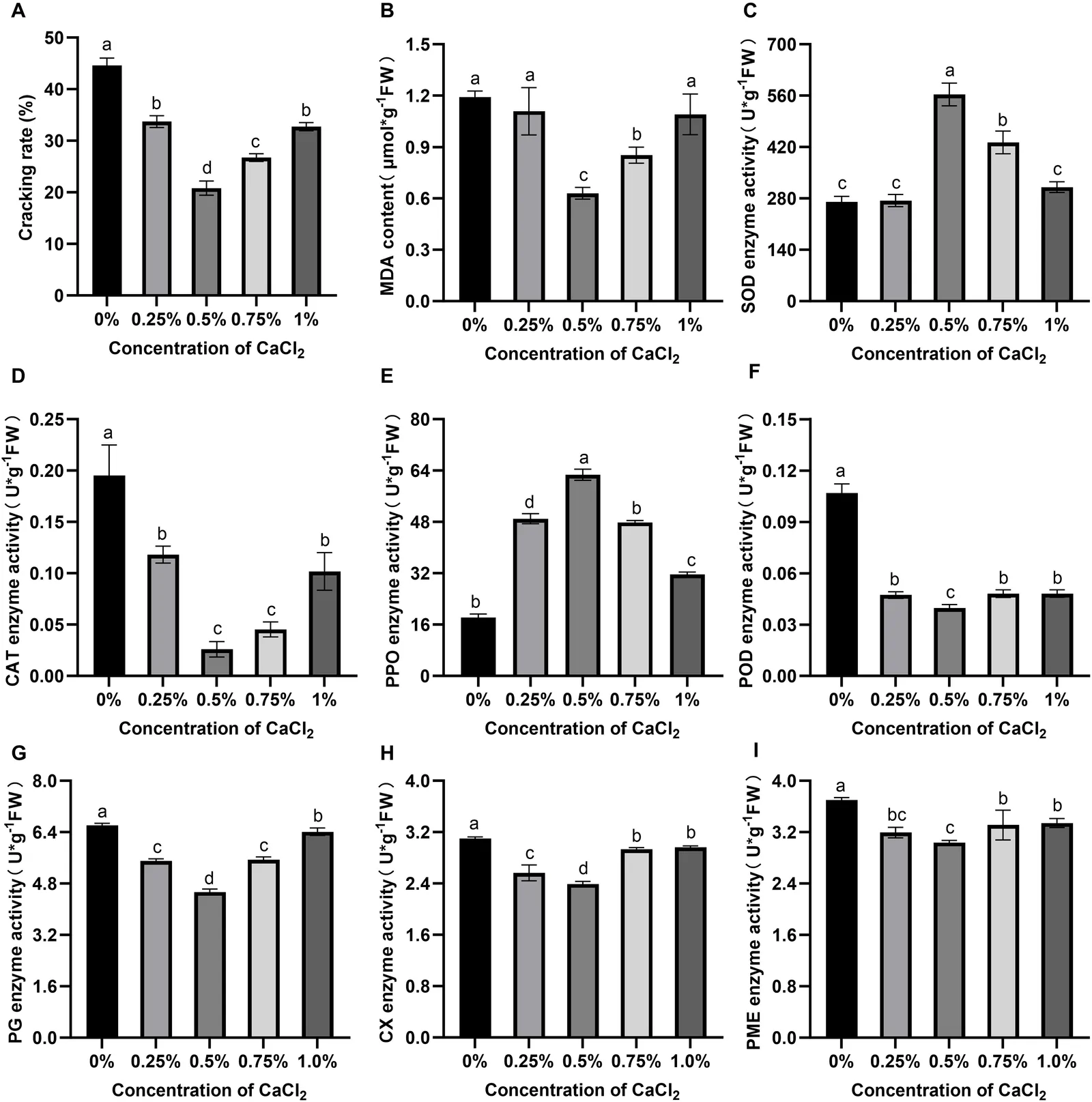
Physiological index differences of sweet cherry under different concent rations of CaCl_2_ treatment. Data are mean ± SE; different letters indicate significant differences at *P*<0.05.

To investigate the effect of exogenous calcium spraying on sweet cherry fruit cracking, the cracking rate and related physiological indicators were measured after treatments with different concentrations of CaCl_2_. The results showed that the 0.25%, 0.5%, 0.75% and 1% CaCl_2_ treatments reduced the cracking rate by 24.4%, 53.3%, 40.0% and 26.6%, respectively, with the 0.5% CaCl_2_ treatment being the most effective, achieving a reduction of 53.3% compared with the control. Under this concentration, fruit MDA content was the lowest, with a decrease of 47.1% compared with the control; SOD and PPO activities were significantly increased, by 108.2% and 245% compared with the control, respectively, while CAT and POD activities were significantly decreased, by 86.7% and 62.8% compared with the control, respectively; cell wall hydrolytic enzymes PG, CX and PME activities all dropped to their lowest levels, decreasing by 31.3%, 22.8% and 17.9% compared with the control, respectively (**Figure 2**). These results indicate that the 0.5% CaCl_2_ treatment effectively alleviates sweet cherry fruit cracking by regulating the antioxidant system and inhibiting cell wall hydrolytic enzyme activities.

### 3.3 Transcriptome analysis for screen candidate genes involved in calcium-regulated fruit cracking

**Figure 3:**
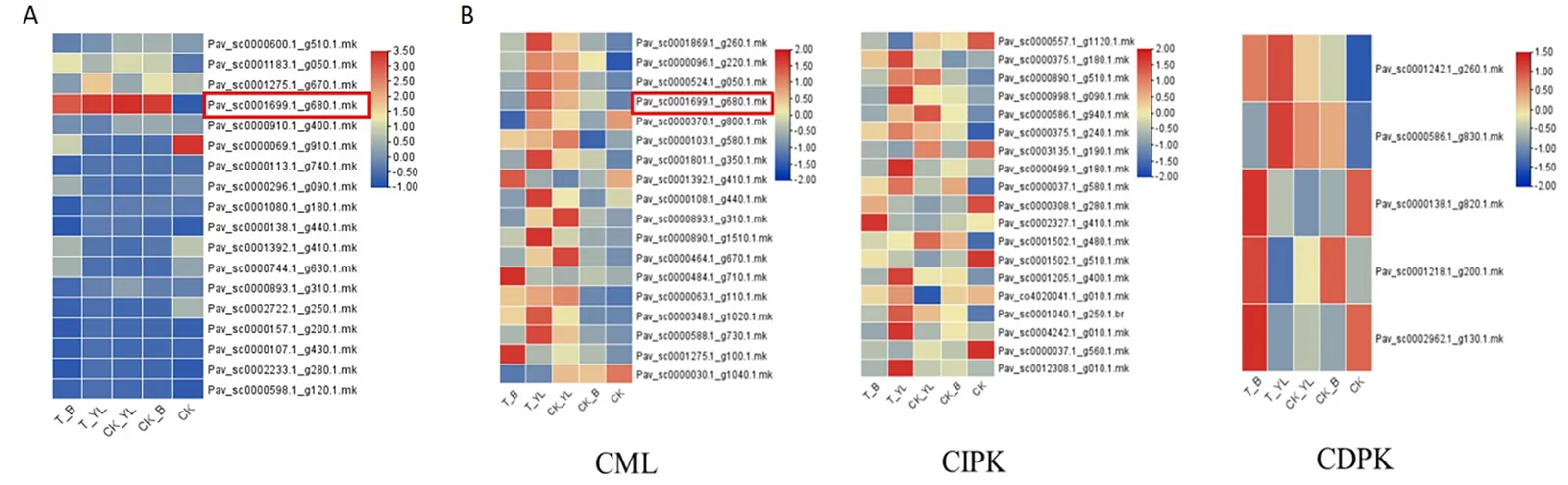
Heatmap analysis of calcium signaling-related differentially expressed genes across different treatment groups. **(A)** Heatmap of the 18 differentially expressed genes. **(B)** Heatmap of calcium signaling-related genes in different treatment groups. CK, untreated intact fruit; CK-B, water-treated non-cracked fruit; CK-YL, cracked tissue from water-treated cracked fruit; T-B, 0.5% CaCl_2_-treated non-cracked fruit; T-YL, cracked tissue from 0.5% CaCl_2_-treated cracked fruit.

To investigate the molecular mechanism by which calcium regulates sweet cherry fruit cracking, transcriptome sequencing analysis was performed on five treatment groups (CK, CK-B, CK-YL, T-B, and T-YL). Using Padj<0.05 and |log2Foldchange|>2 as thresholds for screening differentially expressed genes, 3,068 expressed genes were obtained, from which 18 highly expressed genes related to calcium signaling were further selected (**Table 1**), mainly including members of the CML, CDPK, and CBL families. Heatmap results showed that genes related to calcium signaling had higher expression levels in cracking sites, suggesting that calcium-binding proteins may be involved in the regulation of fruit cracking (**Figure 3B**).

**Table 1:** Calcium signaling-related differentially expressed genes identified in transcriptome analysis.

Gene-id	Gene-description
*Pav-sc0000600.1-g510.1.mk*	calponin homology domain-containing protein DDB-G0272472
*Pav-sc0001183.1-g050.1.mk*	uncharacterized calcium-binding protein At1g02270-like
*Pav-sc0001275.1-g670.1.mk*	calcium uptake protein 1, mitochondrial
*Pav-sc0001699.1-g680.1.mk*	calcium-binding protein CML42
*Pav-sc0000910.1-g400.1.mk*	LOW QUALITY PROTEIN: calcium-dependent protein kinase 2-like
*Pav-sc0000069.1-g910.1.mk*	calcineurin B-like protein 10 isoform X2
*Pav-sc0000113.1-g740.1.mk*	kinesin-like calmodulin-binding protein
*Pav-sc0000296.1-g090.1.mk*	calcium-dependent protein kinase 2
*Pav-sc0001080.1-g180.1.mk*	calcium-binding mitochondrial carrier protein SCaMC-3-like isoform X1
*Pav-sc0000138.1-g440.1.mk*	uncharacterized calcium-binding protein At1g02270
*Pav-sc0001392.1-g410.1.mk*	probable calcium-binding protein CML21
*Pav-sc0000744.1-g630.1.mk*	calreticulin-3-like
*Pav-sc0000893.1-g310.1.mk*	probable calcium-binding protein CML25
*Pav-sc0002722.1-g250.1.mk*	cation/calcium exchanger 2
*Pav-sc0000157.1-g200.1.mk*	calcium-transporting ATPase 12, plasma membrane-type-like
*Pav-sc0000107.1-g430.1.mk*	calcium-dependent protein kinase 26-like
*Pav-sc0002233.1-g280.1.mk*	calcium-transporting ATPase 12, plasma membrane-type-like
*Pav-sc0000598.1-g120.1.mk*	calcium-binding protein PBP1-like

**Figure 4:**
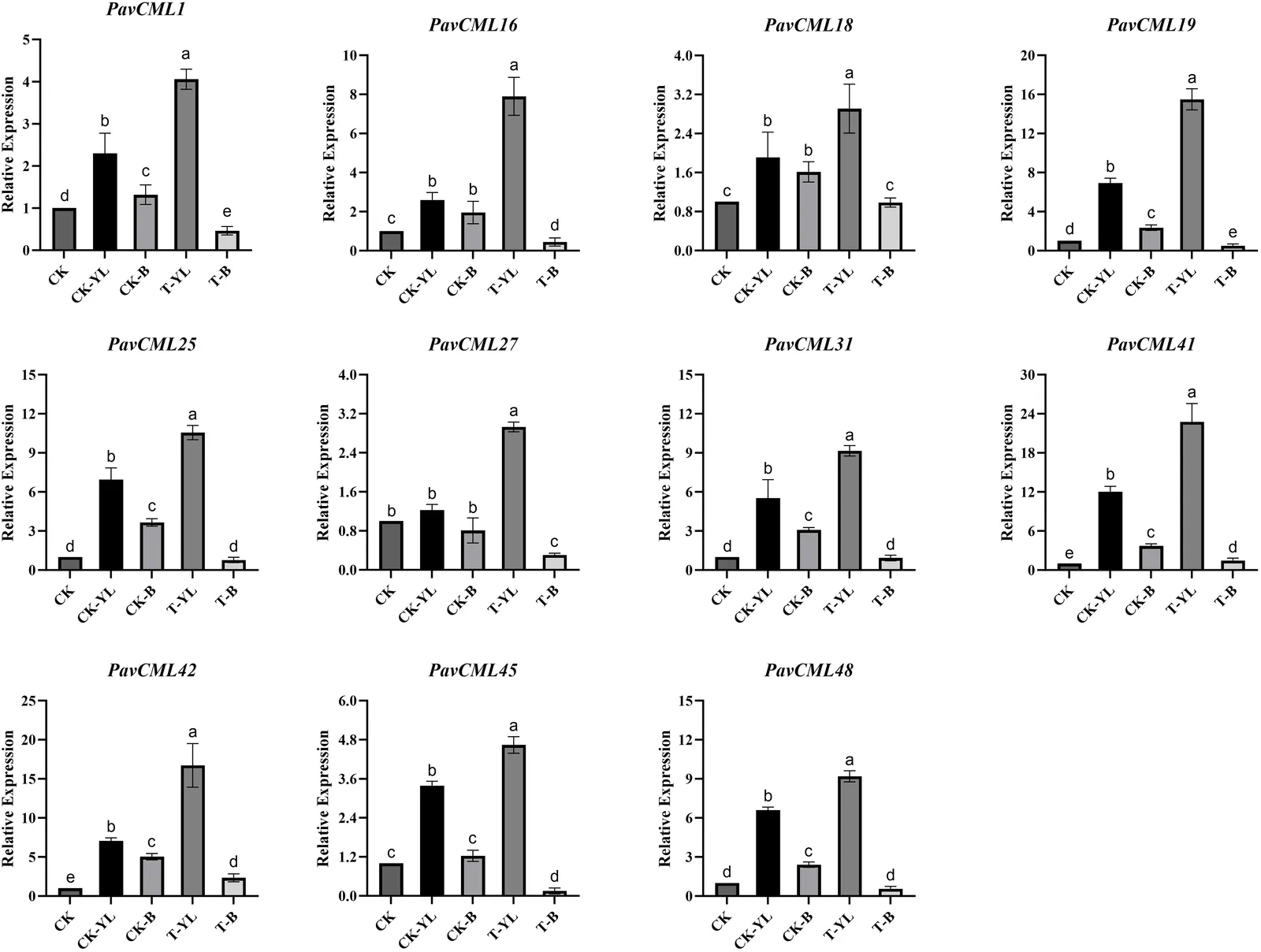
qRT-PCR validation of 11 CML family genes across different treatments. CK, untreated intact fruit; CK-B, water-treated non-cracked fruit; CK-YL, cracked tissue from water-treated cracked fruit; T-B, 0.5% CaCl_2_-treated non-cracked fruit; T-YL, cracked tissue from 0.5% CaCl_2_-treated cracked fruit. Data are mean ± SE. Different letters indicate significant differences at *P* < 0.05.

As shown in **Table 1**, the CML gene family is closely related to calcium-regulated fruit cracking, so 11 CML genes were randomly selected for qRT-PCR validation. The results were consistent with the trends in the transcriptome sequencing data, confirming the reliability of the transcriptome data (**Figure 4**). Further analysis showed that the expression levels of members of this gene family were significantly increased in water-sprayed cracked fruits (CK-YL) and calcium-sprayed cracked fruits (T-YL) compared with CK, and the relative expression in calcium-sprayed cracked fruits was higher than in water-sprayed fruits, suggesting that this gene family is involved in regulating the fruit cracking process in sweet cherry. Among them, *PavCML42* showed the most significant differential expression and was selected as a candidate gene for subsequent functional validation (**Figure 3A**).

### 3.4 Preliminary exploration of the *PavCML42* gene function

#### 3.4.1 Analysis of tissue-specific expression and developmental stage expression

**Figure 5:**
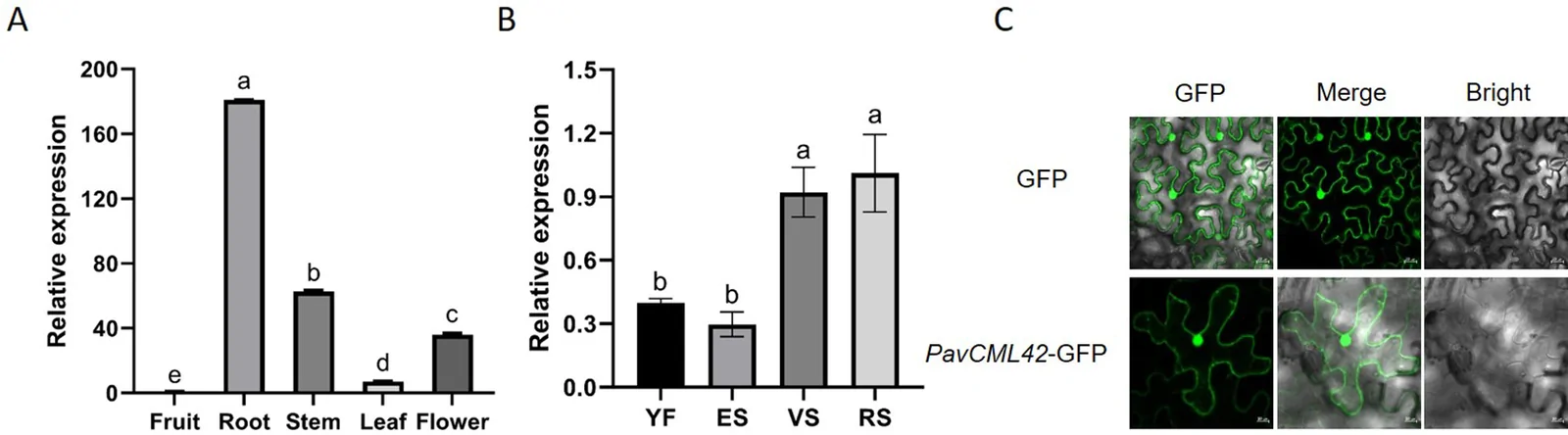
Expression patterns and subcellular localization of *PavCML42*. **(A)** Tissue-specific expression of *PavCML42* in root, stem, leaf, flower, and fruit. **(B)** Expression dynamics at four fruit developmental stages: YF (young fruit), ES (expansion stage), VS (veraison), and RS (ripening). **(C)** Subcellular localization of *PavCML42* in tobacco leaf epidermal cells. For **(A, B)**, data are mean ± SE; different letters indicate significant differences at *P* < 0.05.

qRT-PCR results showed that the expression levels of *PavCML42* varied significantly among different tissues and organs of sweet cherry. Among the tissues examined, the highest expression was observed in roots, followed by stems and flowers, with a significant difference between the latter two. Expression in leaves was relatively low, and the lowest level was detected in fruits (**Figure 5A**). This indicated that *PavCML42* expression exhibited pronounced tissue specificity. Its high expression in roots suggested that this gene might be involved in calcium signal perception and transduction in the root system, while its low expression in fruits implied that it might be induced and activated only at specific developmental stages or under stress conditions.

We further examined the expression dynamics of *PavCML42* across different developmental stages of sweet cherry fruits. The results showed that its expression level was relatively low at the young fruit and expansion stages, but increased significantly at veraison and reached the highest level at the ripening stage (**Figure 5B**). This expression pattern is consistent with the physiological changes occurring during fruit ripening, including active cell wall metabolism, reduced peel mechanical strength, and increased susceptibility to cracking, further supporting the involvement of *PavCML42* in cracking regulation at the ripening stage.

#### 3.4.2 Subcellular localization analysis of *PavCML42* in tobacco

Confocal microscopy observation revealed that the fluorescence signal of the pZP211-GFP empty vector control was diffusely and evenly distributed throughout the entire cell, with no obvious organelle enrichment. In contrast, the fluorescence signal of the *PavCML42*-GFP fusion protein was mainly concentrated at the cell periphery and in the nuclear region, consistent with the morphological characteristics of the plasma membrane and the nucleus (**Figure 5C**). This result indicated that the *PavCML42* protein is localized to the plasma membrane and the nucleus, supporting its functional role as a calcium signal sensor—its plasma membrane localization facilitates the perception of extracellular calcium signal changes, while its nuclear localization may be involved in the transcriptional regulation of downstream target genes.

#### 3.4.3 Transient overexpression of *PavCML42*

**Figure 6:**
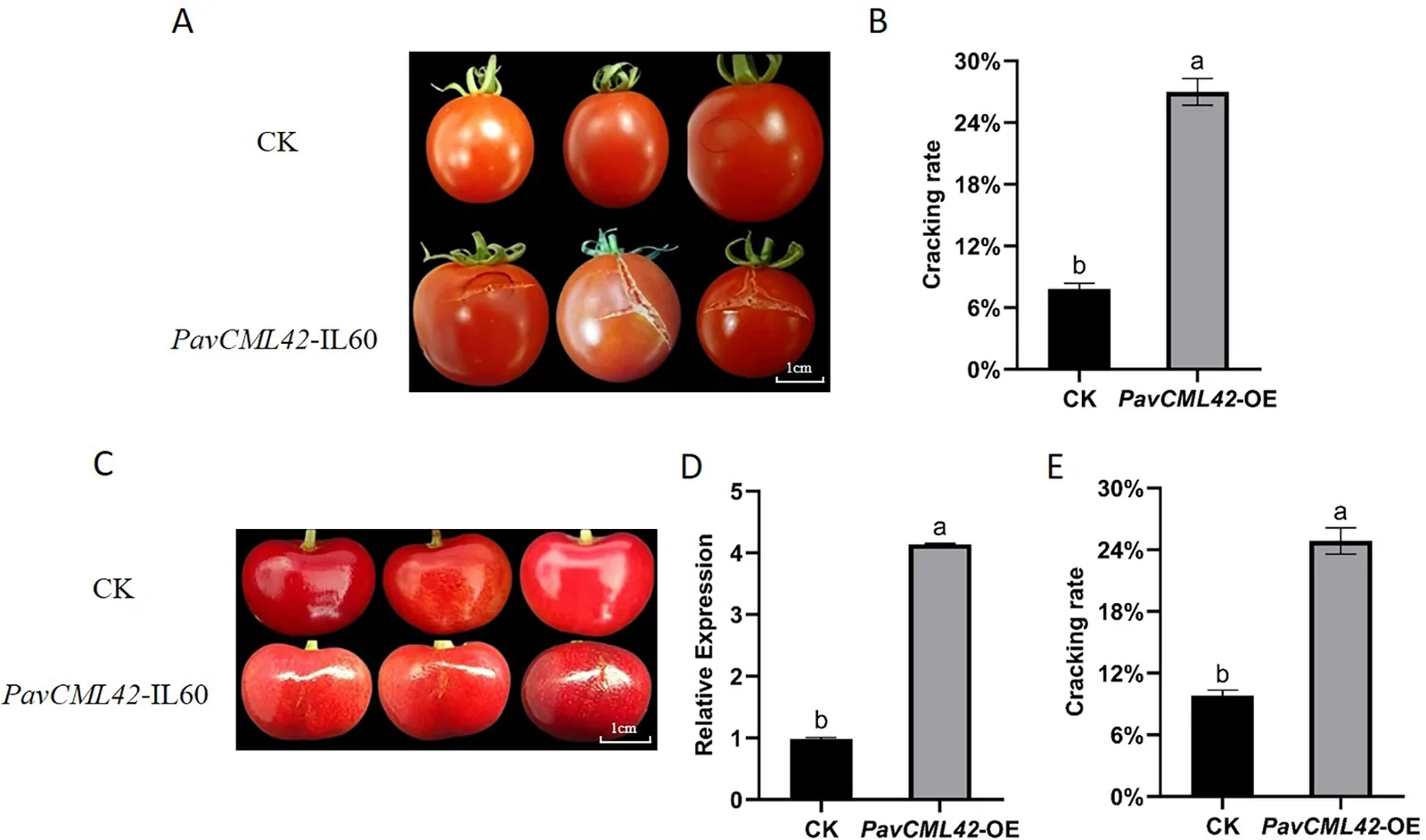
Phenotypic and molecular characterization of *PavCML42* overexpression in tomato and sweet cherry fruits. **(A, B)** Phenotypes and cracking rates of tomato fruits; **(C–E)** Phenotypes, expression levels, and cracking rates of sweet cherry fruits. Data are mean ± SE; different letters indicate significant differences at *P* < 0.05.

**Figure 7:**
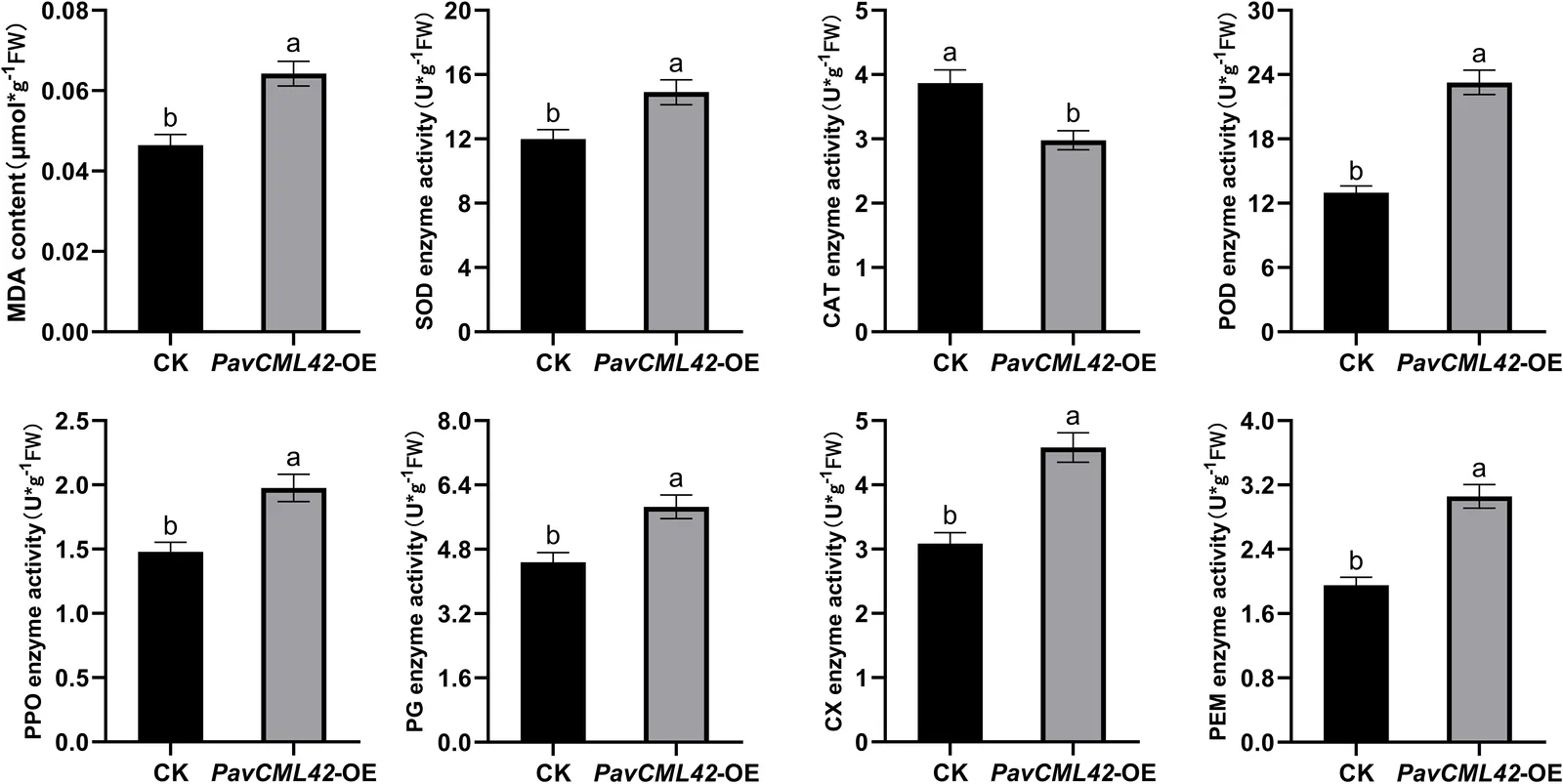
Physiological measurements of sweet cherry after transient overexpression of *PavCML42*. Data are mean ± SE. Different letters indicate significant differences at *P* < 0.05.

To verify the role of *PavCML42* in fruit cracking regulation, an IL60-*PavCML42* overexpression vector was constructed and used for transient overexpression experiments in tomato and sweet cherry fruits. In tomato fruits, the empty vector control group exhibited a cracking rate of approximately 7.8% at the injection sites, reflecting the background physical damage caused by the injection procedure itself. In contrast, injection of IL60-*PavCML42* significantly increased the cracking rate to approximately 27.0%, representing an increase of about 19.2 percentage points over the control (**Figures 6A, B**), with the difference being highly significant (*P* < 0.01). In sweet cherry fruits, qRT-PCR confirmed that *PavCML42* expression at the injection sites was significantly upregulated (**Figures 6C, D**), and the cracking rate of fruits overexpressing *PavCML42* was 15% higher than that of the empty vector control (**Figure 6E**). Physiological measurements showed that *PavCML42* overexpression significantly increased the activities of cell wall–degrading enzymes (PG, CX, PME), elevated MDA content, and increased SOD, CAT, and PPO activities (**Figure 7**). These results indicate that, after excluding the background damage caused by injection, overexpression of *PavCML42* promotes cell wall degradation, exacerbates membrane lipid peroxidation, and consequently induces fruit cracking.

#### 3.4.4 Gene silencing of *PavCML42*

**Figure 8:**
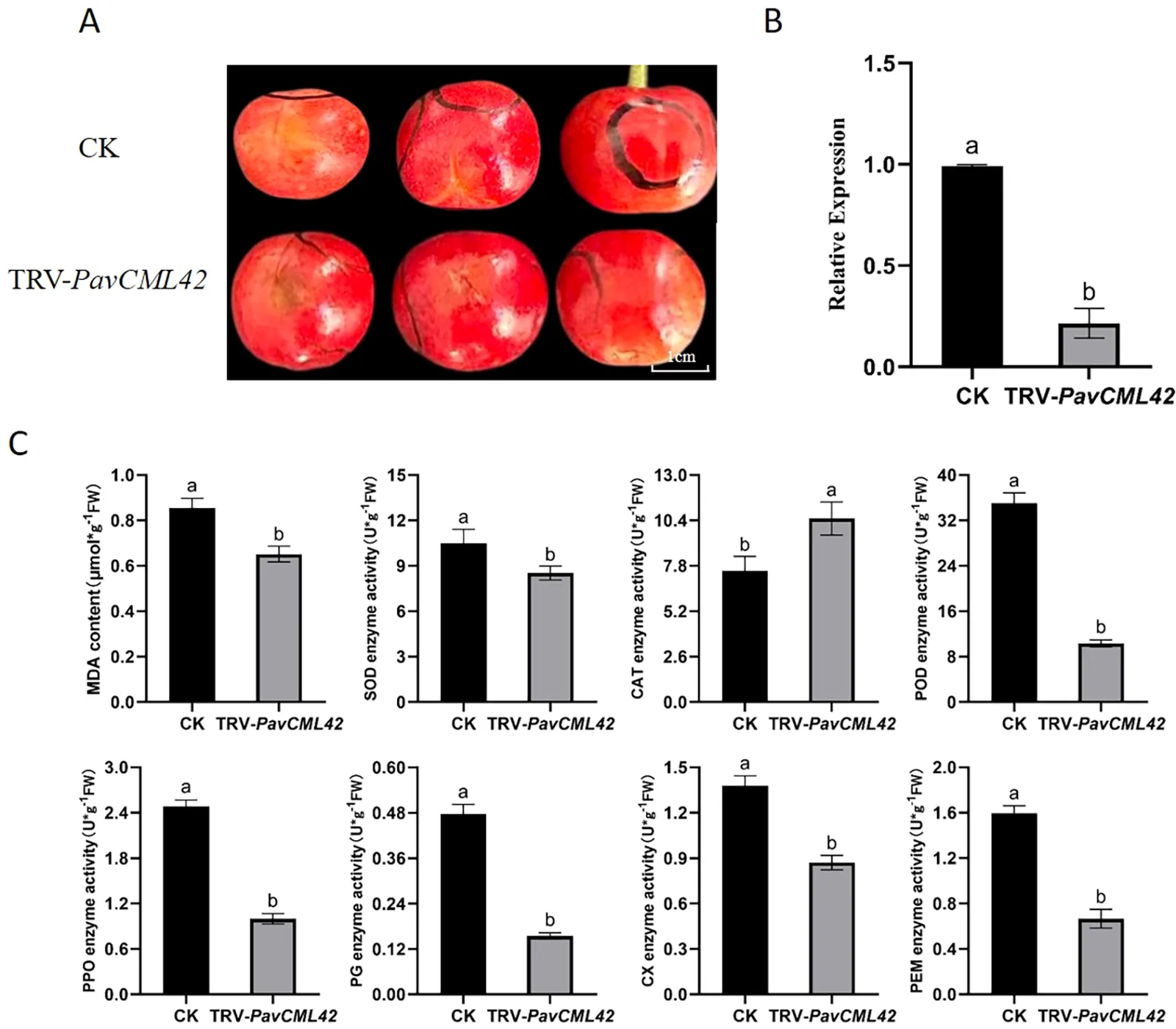
Phenotypic and physiological characterization of *PavCML42* silencing in sweet cherry fruits. **(A)** Phenotypes; **(B)** Relative expression levels; **(C)** Physiological indices. Data are mean ± SE. Different letters indicate significant differences at *P* < 0.05.

A TRV2-*PavCML42* silencing vector was constructed, and *PavCML42* was silenced in sweet cherry fruit using the VIGS technique (**Figure 8A**). qRT-PCR analysis showed that, compared with the control, the expression level of *PavCML42* at the TRV2-*PavCML42* injection sites was significantly reduced, achieving a silencing efficiency of 78.2% (**Figure 8B**). Physiological measurements indicated that after silencing *PavCML42*, the activities of cell wall hydrolases(PG, CX, PME) and antioxidant enzymes (SOD, POD, PPO) were all significantly decreased, and MDA accumulation was reduced (**Figure 8C**). This results further support the involvement of *PavCML42* in the fruit cracking process in sweet cherry, as silencing this gene reduces the activity of cracking-related enzymes and alleviates membrane lipid peroxidation damage.

## 4 Discussion

Rain-induced fruit cracking during sweet cherry ripening is one of the main factors limiting industry development. In this study, using exogenous calcium spraying, physiological measurements, transcriptome analysis, and gene function validation, we systematically explored the physiological and molecular mechanisms by which calcium regulates sweet cherry cracking. The results show that 0.5% CaCl_2_ treatment significantly reduced the cracking rate, modulated the balance of the antioxidant system, and inhibited cell wall hydrolytic enzyme activities. Transcriptome analysis identified multiple differentially expressed genes related to calcium signaling, among which *PavCML42* was highly expressed in cracked fruits. Transient overexpression of *PavCML42* promoted cracking, while gene silencing reduced the activities of crack-related enzymes, further suggesting its involvement in the cracking process. The detached-fruit submersion assay was used to quantify the intrinsic physiological effects of calcium on fruit epidermal mechanical strength under controlled conditions (Winkler et al., 2024). and is widely adopted in cracking studies of cherry, grape, and tomato (Wang et al., 2016; Lichter et al., 2002).

Consistent with a previous study on sweet cherry (Deniz, 2014), exogenous application of 0.5% CaCl_2_ significantly reduced the cracking rate in the present study. However, that study only focused on changes in crack rate and did not address regulation of cell wall metabolic enzyme activities. In our study, fruits treated with this concentration accumulated the least MDA and had the highest SOD and PPO activities, while CAT, POD, and cell wall hydrolytic enzyme activities decreased to their lowest levels. The inhibitory effect of calcium on cell wall hydrolytic enzymes has been reported in various fruit trees, including lychee (Peng et al., 2001), apple (Idit and Stern, 2019; Ortiz et al., 2011), Chinese cherry (Wu et al., 2023), and nectarine (Zhu et al., 2023), suggest that calcium-mediated inhibition of cell wall hydrolytic enzymes is conserved across species.

To investigate the physiological basis of cracking, this study compared relevant indicators between cracked and normal fruits. Results showed that SOD and CAT activities in the peel of cracked fruits were lower than those in normal fruits, whereas POD, PPO, PG, and CX activities were significantly increased, accompanied by decreased protopectin content and increased soluble pectin content. These results are consistent with the findings of Wen and Shi (2012) in Jincheng orange and Wang et al. (2013) in Huping jujube, indicating that increased cell wall hydrolytic enzyme activity and pectin degradation are important physiological causes of fruit cracking. However, PME activity did not differ significantly between cracked and normal fruits, suggesting that PG and CX may play more important roles in sweet cherry cracking.

Using transcriptome analysis, this study identified 18 differentially expressed genes related to calcium signaling, among which members of the CML family were predominant and *PavCML42* showed the most significant differential expression; therefore, it was selected as a candidate gene for functional studies. Tissue-specific expression analysis showed that *PavCML42* had the highest expression in roots, followed by stems, flowers, and leaves, with the lowest expression in fruits. Further examination of its expression during fruit development revealed that *PavCML42* showed the highest transcript level in ripening fruits, which is precisely the stage when sweet cherry is most susceptible to cracking, suggesting that this gene may be closely associated with the regulation of cracking at the ripening stage. To verify the role of *PavCML42* in cracking regulation, transient overexpression experiments were conducted in tomato and sweet cherry fruits. The results showed that overexpression of *PavCML42* in both tomato and sweet cherry fruits exhibited pronounced fruit cracking phenotypes at the injection sites, with a significant increase in cracking rate; qRT-PCR confirmed successful up-regulation of the target gene. Physiological measurements showed that *PavCML42* overexpression significantly increased cell wall hydrolytic enzyme activity. This finding is consistent with that of Brummell and Harpster (2001) in tomato, where increased PG activity enhances peel softening and susceptibility to cracking. At the same time, overexpression exacerbated membrane lipid peroxidation; the concurrent increase in antioxidant enzyme activities may be a stress response of the fruit to oxidative damage. Interestingly, exogenous calcium treatment reduced the cracking rate while upregulating *PavCML42* expression. This apparent paradox can be explained as follows: calcium reduces cracking primarily through physical cross-linking with pectin to form calcium bridges, reinforcing peel structure independently of *PavCML42* transcription (Winkler et al., 2024); the upregulation of *PavCML42* likely represents a compensatory response to calcium-induced perturbation of calcium homeostasis (Medvedev, 2005). Therefore, our conclusion that *PavCML42* functions is involved in cracking regulation is based on three lines of evidence—high expression in cracked tissues, overexpression-induced cracking, and silencing-suppressed cracking—rather than on its whole-fruit expression trend under calcium treatment.

*PavCML42* silencing further confirmed its function: silencing significantly reduced cell wall hydrolytic enzyme activities and membrane lipid peroxidation levels, corroborating the overexpression results. In recent years, the functions of CML family members in cell wall metabolism and fruit cracking regulation have been gradually revealed. In pomegranate, *PgCML23* was significantly upregulated under bagging treatment, and its heterologous expression in tomato significantly reduced the fruit cracking rate by strengthening the cell wall (Wang et al., 2024). In papaya, the calcium sensor protein *CpCML46* interacts with the transcriptional repressor *CpERF12* to relieve its inhibition of cell wall degradation-related genes, thereby positively regulating fruit ripening and cell wall degradation (Zhu et al., 2026). In jujube, *CJCML19* showed medium-to-low expression in cracked fruit skins and may be involved in regulating water entry and exit, potentially contributing to cracking resistance (Lin et al., 2021). The functions of *CML42* genes have diversified among different species: *Arabidopsis AtCML42* participates in drought response by inhibiting ABA synthesis (Vadassery et al., 2012), rice *OsCML4* enhances drought tolerance (Yin et al., 2015), and tea tree *CsCML42* responds to salt stress (Ma et al., 2019). This study found that *PavCML42* participates in fruit cracking regulation, differs from the functions reported for *CML42* homologs in other species. Such functional divergence may be related to differences in the number of EF-hand domains, tissue-specific expression patterns, and downstream interacting proteins, suggesting that *CML42* may have evolved distinct regulatory mechanisms in different species.

As a second messenger, calcium ions are sensed and transduce intracellular calcium concentration changes through calcium-binding proteins (Medvedev, 2005). In this study, exogenous calcium treatment may have increased intracellular Ca^2+^ concentration, activating calcium signal–related proteins represented by *PavCML42*, thereby initiating defense mechanisms such as cell wall reinforcement; once calcium homeostasis was restored, *PavCML42* expression was downregulated to avoid excessive energy consumption. This speculation is similar to the regulatory mechanism reported for *PavCBL4* induction under salt stress (Fu et al., 2023). It is noteworthy that, in addition to CMLs, the transcriptome data in this study also identified differential expression of CDPK and CBL family members. CMLs have simple structures and mainly act as early responders to calcium signals; CDPKs combine calcium-binding and kinase activities and can directly phosphorylate downstream target proteins (Schulz et al., 2013); CBLs must interact with CIPKs to form complexes to become functional (Weinl and Kudla, 2009). Due to structural differences among the three protein types, it is inferred that they may occupy different hierarchical levels in calcium signal transduction and cooperatively regulate downstream physiological processes.

In summary, we identified *PavCML42* as a gene involved in fruit cracking regulation in sweet cherry through transient overexpression and VIGS assays. Our results demonstrate that *PavCML42* participates in cracking by enhancing cell wall hydrolase activities and exacerbating membrane lipid peroxidation, providing a candidate gene for understanding calcium-mediated cracking regulation in sweet cherry. These findings lay a foundation for further dissecting the calcium signaling network underlying fruit cracking in woody plants.

## Data Availability

The original contributions presented in the study are included in the article/**Supplementary Material**, further inquiries can be directed to the corresponding author.

## Supplementary material

Supplementary Table 1 Primers used for qRT-PCR and vector construction.

Supplementary Table 2 Sequencing quality statistics of ‘Brooks’ fruits.
